# Poly[[diaqua­bis(μ_2_-4,4′-bipyridine)­manganese(II)] bis­[2-(2-carboxy­phenyl­di­sulfanyl)benzoate]]

**DOI:** 10.1107/S1600536809013646

**Published:** 2009-04-22

**Authors:** Min Hu, Song-Tao Ma, Liang-Qi Guo, Shao-Ming Fang

**Affiliations:** a Zhengzhou University of Light Industry, Henan Provincial Key Laboratory of Surface and Interface Science, Henan, Zhengzhou 450002, People’s Republic of China

## Abstract

The title complex, {[Mn(C_10_H_8_N_2_)_2_(H_2_O)_2_](C_14_H_9_O_4_S_2_)_2_}_*n*_, contains an octa­hedrally coordinated Mn^II^ cation and 2-(2-carboxy­phenyl­disulfan­yl)benzoate anions. The Mn^II^ center is situated on a crystallographic center of inversion and is coordinated by four 4,4′-bipyridine (4,4′-bipy) ligands and two water mol­ecules. The 4,4′-bipy ligands act as bridging ligands, producing a fishing-net-like two-dimensional framework. In the crystal structure, this positively charged framework is charge balanced by 2-(2-carboxy­phenyl­disulfan­yl)benzoate anions that form a separate anionic two-dimensional framework *via* inter­molecular O—H⋯O hydrogen bonds and C—H⋯π stacking inter­actions. Additional inter­molecular O—H⋯O hydrogen bonds link the cationic and anionic frameworks to form the three-dimensional crystal structure.

## Related literature

For general background on the design and synthesis of coordination polymers, see: James (2003 ▶); Kitagawa *et al.* (2004 ▶); Steel (2005 ▶); Ye *et al.* (2005 ▶). For the crystal structures of related complexes with 4,4′-bipyridine ligands, see: Biradha *et al.* (2006 ▶); Denning *et al.* (2008 ▶); Hoffart *et al.* (2007 ▶); Noro *et al.* (2002 ▶); Qin *et al.* (2007 ▶); Zhang *et al.* (2007 ▶). For metal–organic framework materials containing 2,2′-dithio­dibenzoic acid, see: Humphrey *et al.* (2004 ▶); Murugavel *et al.* (2001 ▶); Wang *et al.* (2004 ▶); Zhao *et al.* (2004 ▶).
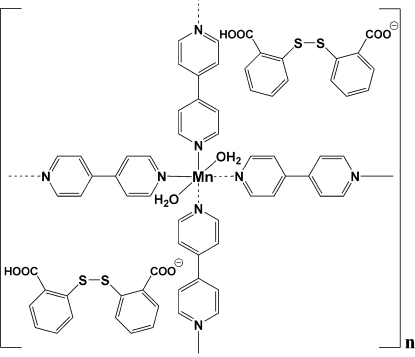


## Experimental

### 

#### Crystal data

[Mn(C_10_H_8_N_2_)_2_(H_2_O)_2_](C_14_H_9_O_4_S_2_)_2_
*M* *_r_* = 1014.00
Triclinic, 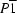

*a* = 8.260 (5) Å
*b* = 11.771 (7) Å
*c* = 11.917 (7) Å
α = 94.334 (6)°
β = 102.339 (7)°
γ = 96.217 (7)°
*V* = 1119 (1) Å^3^
*Z* = 1
Mo *K*α radiation
μ = 0.55 mm^−1^
*T* = 293 K
0.41 × 0.13 × 0.09 mm

#### Data collection

Bruker SMART CCD area-detector diffractometer
Absorption correction: multi-scan (*SADABS*; Sheldrick, 1996 ▶) *T*_min_ = 0.807, *T*_max_ = 0.952
8248 measured reflections
3921 independent reflections
3242 reflections with *I* > 2σ(*I*)
*R*_int_ = 0.018

#### Refinement

*R*[*F* ^2^ > 2σ(*F* ^2^)] = 0.032
*wR*(*F* ^2^) = 0.081
*S* = 1.03
3921 reflections
304 parameters
H-atom parameters constrained
Δρ_max_ = 0.24 e Å^−3^
Δρ_min_ = −0.21 e Å^−3^

### 

Data collection: *SMART* (Bruker, 1998 ▶); cell refinement: *SAINT* (Bruker, 1998 ▶); data reduction: *SAINT*; program(s) used to solve structure: *SHELXS97* (Sheldrick, 2008 ▶); program(s) used to refine structure: *SHELXL97* (Sheldrick, 2008 ▶); molecular graphics: *SHELXTL* (Sheldrick, 2008 ▶); software used to prepare material for publication: *SHELXTL* and *PLATON* (Spek, 2009 ▶).

## Supplementary Material

Crystal structure: contains datablocks I, global. DOI: 10.1107/S1600536809013646/im2106sup1.cif

Structure factors: contains datablocks I. DOI: 10.1107/S1600536809013646/im2106Isup2.hkl

Additional supplementary materials:  crystallographic information; 3D view; checkCIF report

## Figures and Tables

**Table 1:** Hydrogen-bond geometry (Å, °)

*D*—H⋯*A*	*D*—H	H⋯*A*	*D*⋯*A*	*D*—H⋯*A*
O1—H11⋯O2	0.85	1.92	2.761 (3)	173
O1—H12⋯O3^i^	0.85	1.82	2.667 (3)	174
O5—H51⋯O2^ii^	0.82	1.83	2.637 (3)	169
C4—H4⋯S1^iii^	0.93	2.86	3.562 (3)	133
C23—H23⋯S2	0.93	2.66	3.191 (3)	117
C22—H22⋯*Cg*1^iv^	0.93	2.94	3.79 (2)	153

Symmetry codes: (i) 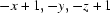; (ii) 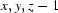; (iii) 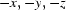; (iv) 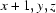. *Cg*1 is the centroid of the C12–C17 ring.

**Fig. 1.**
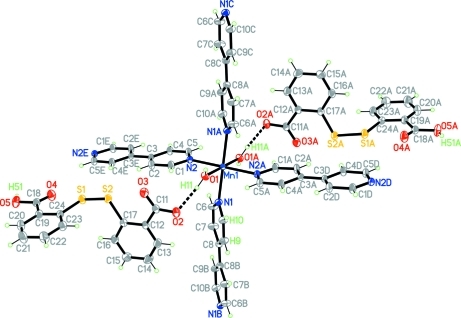
The molecular structure of (I), showing the atom-labelling scheme and the intermolecular O–H···O hydrogen-bonding interactions. Displacement ellipsoids are drawn at the 30% probability level. The atoms labelled with the suffixes A, B, C, D, and E are generated by the symmetry operations (–x, –y, 1 –z), (–x, –y + 1, –z + 1), (–x, y - 1, z), (x, y, z + 1), and (–x, –y, –z), respectively.

**Fig. 2.**
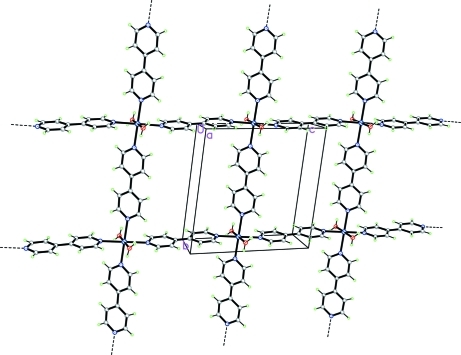
Cationic two-dimensional network, parallel to the (001) plane, formed by [Mn(4,4'-bipy)4(H2O)2] units.

**Fig. 3.**
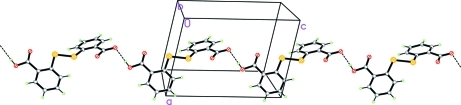
Anionic two-dimensional network, parallel to the (100) plane, formed by intermolecular O—H···O hydrogen bonds and C—H···π stacking interactions between 2-o-benzoato-disulfanyl-benzoic acid anions.

## supplementary crystallographic information

### Comment

Rational design and synthesis of novel coordination polymers have achieved considerable progress in the field of supramolecular chemistry and crystal engineering. (James, 2003; Kitagawa *et al.*, 2004; Steel, 2005; Ye *et al.*, 2005). Particularly classical ligands are represented such as 4,4' -bipy. Numerous polymeric transition metal complexes with 4,4'-bipy have been synthesized and structurally characterized to date. 4,4'-bipyridine is an ideal connector between transition metal atoms for the propagation of coordination networks due to its two potential binding sites (Denning *et al.*, 2008; Hoffart *et al.*, 2007; Zhang *et al.*, 2007; Qin *et al.*, 2007; Noro *et al.*, 2002; Biradha *et al.*, 2006). During our investigation of the assembly of metal ions and mixed ligands (4,4'-bipyridine and 2,2'-dithiodibenzoic acid), we did not obtain the expected compound. The title complex,{[Mn(C_10_H_8_N_2_)_2_(H_2_O)_2_] (C_14_H_9_O_4_S_2_)_2_}_n_, (I) was synthesized. In contrast to many metal-organic framework materials containing 2,2'-dithiodibenzoic acid, the anions in I do not act as bridging ligands, but rather as extraframework guest molecules (Murugavel *et al.*, 2001; Zhao *et al.*, 2004; Humphrey *et al.*, 2004; Wang *et al.*, 2004). In the title crystal structure, each Mn^II^ center is separated by 4,4'-bipy spacers to give a large rhombic arrangement with each metal ion adopting an octahedral environment by binding to four 4,4' -bipy ligands and two water molecules (Fig. 1). All of the 4,4'-bipy molecules act as doubly bridging ligands. Mn^II^ centers are bridged by 4,4'-bipy ligands to form a fishing-net like, two-dimensional framework (Fig.2). In the crystal structure, the positively charged framework of I is charge balanced by 2-*o*-benzoato-disulfanyl-benzoic acid anions. Intermolecular O—H···O hydrogen bonds and C—H··· π stacking interactions (Table 1) (Fig.3) between anions link them to form a two-dimensional framework. Intermolecular O—H···O hydrogen bonds link the cationic and anionic frameworks (Fig.1, Table 1) to form the observed crystal structure.

### Experimental

A solution of 4,4'-bipy (0.05 mmol) and 2,2'-dithiodibenzoic acid (0.05 mmol) in CH_3_OH (10 ml) in the presence of excess 2,6-dimethylpyridine (*ca.* 0.05 ml for adjusting the pH value of the reaction system to basic conditions) was carefully layered on top of an aqueous solution (15 ml) of Mn(ClO_4_)_2 _(0.1 mmol) in a test tube. Yellow single crystals suitable for X-ray analysis appeared at the tube wall after *ca.* one month at room temperature (yield ~30% based on 4,4'-bipy). Elemental analysis calculated for (C_48_H_38_MnN_4_O_10_S_4_): H 3.78 C 56.86 N 5.53%; found: H 3.52, C 56.62, N 5.34%. IR (KBr pellet, cm^-1^): 3094br, 1690*m*, 1594vs, 1540 s, 1457*m*, 1412*m*, 1377 s, 1306w, 1258 s, 1232*m*, 1171w, 1144*m*, 1037*m*,1000w, 903w, 806*m*, 742 s, 689*m*, 649w, 622*m*, 542w, 468w.

### Refinement

H atoms were included in calculated positions and treated in the subsequent refinement as riding atoms, with C—H = 0.93 Å and O—H = 0.82 (carboxylic) or 0.85 Å (water), and with Uiso(H) = 1.2Ueq(C) and 1.5Ueq(O).

### Crystal data

**Table:**

[Mn(C_10_H_8_N_2_)_2_(H_2_O)_2_](C_14_H_9_O_4_S_2_)_2_	*Z* = 1
*M**_r_* = 1014.00	*F*(000) = 523
Triclinic, *P*1	*D*_x_ = 1.504 Mg m^−^^3^
Hall symbol: -P 1	Mo *K*α radiation, λ = 0.71073 Å
*a* = 8.260 (5) Å	Cell parameters from 3063 reflections
*b* = 11.771 (7) Å	θ = 2.6–27.3°
*c* = 11.917 (7) Å	µ = 0.55 mm^−^^1^
α = 94.334 (6)°	*T* = 293 K
β = 102.339 (7)°	Prism, yellow
γ = 96.217 (7)°	0.41 × 0.13 × 0.09 mm
*V* = 1119 (1) Å^3^	

### Data collection

**Table:**

Bruker SMART CCD area-detector diffractometer	3921 independent reflections
Radiation source: fine-focus sealed tube	3242 reflections with *I* > 2σ(*I*)
graphite	*R*_int_ = 0.018
φ and ω scans	θ_max_ = 25.0°, θ_min_ = 2.5°
Absorption correction: multi-scan (*SADABS*; Sheldrick, 1996)	*h* = −9→9
*T*_min_ = 0.807, *T*_max_ = 0.952	*k* = −14→14
8248 measured reflections	*l* = −14→13

### Refinement

**Table:**

Refinement on *F*^2^	Primary atom site location: structure-invariant direct methods
Least-squares matrix: full	Secondary atom site location: difference Fourier map
*R*[*F*^2^ > 2σ(*F*^2^)] = 0.032	Hydrogen site location: inferred from neighbouring sites
*wR*(*F*^2^) = 0.081	H-atom parameters constrained
*S* = 1.03	*w* = 1/[σ^2^(*F*_o_^2^) + (0.0338*P*)^2^ + 0.4902*P*] where *P* = (*F*_o_^2^ + 2*F*_c_^2^)/3
3921 reflections	(Δ/σ)_max_ = 0.001
304 parameters	Δρ_max_ = 0.24 e Å^−^^3^
0 restraints	Δρ_min_ = −0.21 e Å^−^^3^

### Special details

**Table:**

Geometry. All e.s.d.'s (except the e.s.d. in the dihedral angle between two l.s. planes) are estimated using the full covariance matrix. The cell e.s.d.'s are taken into account individually in the estimation of e.s.d.'s in distances, angles and torsion angles; correlations between e.s.d.'s in cell parameters are only used when they are defined by crystal symmetry. An approximate (isotropic) treatment of cell e.s.d.'s is used for estimating e.s.d.'s involving l.s. planes.
Refinement. Refinement of *F*^2^ against ALL reflections. The weighted *R*-factor *wR* and goodness of fit *S* are based on *F*^2^, conventional *R*-factors *R* are based on *F*, with *F* set to zero for negative *F*^2^. The threshold expression of *F*^2^ > σ(*F*^2^) is used only for calculating *R*-factors(gt) *etc*. and is not relevant to the choice of reflections for refinement. *R*-factors based on *F*^2^ are statistically about twice as large as those based on *F*, and *R*- factors based on ALL data will be even larger.

### Fractional atomic coordinates and isotropic or equivalent isotropic displacement parameters (Å^2^)

**Table:**

	*x*	*y*	*z*	*U*_iso_*/*U*_eq_	
Mn1	0.0000	0.0000	0.5000	0.02618 (12)	
C1	0.1323 (3)	0.0099 (2)	0.26331 (18)	0.0413 (5)	
H1	0.2313	0.0235	0.3195	0.050*	
C2	0.1417 (3)	0.0171 (2)	0.14994 (18)	0.0426 (6)	
H2	0.2450	0.0358	0.1321	0.051*	
C3	−0.0016 (2)	−0.00339 (17)	0.06212 (16)	0.0288 (4)	
C4	−0.1492 (3)	−0.03266 (19)	0.09752 (17)	0.0367 (5)	
H4	−0.2496	−0.0490	0.0428	0.044*	
C5	−0.1486 (3)	−0.03779 (19)	0.21322 (17)	0.0365 (5)	
H5	−0.2499	−0.0580	0.2333	0.044*	
C6	−0.0798 (3)	0.24339 (19)	0.4099 (2)	0.0470 (6)	
H6	−0.1457	0.1943	0.3484	0.056*	
C7	−0.0917 (3)	0.35907 (19)	0.4094 (2)	0.0499 (6)	
H7	−0.1659	0.3853	0.3497	0.060*	
C8	0.0067 (3)	0.43706 (17)	0.49789 (18)	0.0329 (5)	
C9	0.1167 (3)	0.38947 (19)	0.58152 (19)	0.0420 (5)	
H9	0.1880	0.4370	0.6419	0.050*	
C10	0.1207 (3)	0.27247 (19)	0.57538 (19)	0.0418 (5)	
H10	0.1977	0.2441	0.6318	0.050*	
C11	0.4897 (3)	0.19836 (19)	0.37959 (18)	0.0366 (5)	
C12	0.4734 (3)	0.29917 (18)	0.30933 (17)	0.0344 (5)	
C13	0.4283 (3)	0.3990 (2)	0.3568 (2)	0.0488 (6)	
H13	0.3984	0.3993	0.4278	0.059*	
C14	0.4264 (4)	0.4974 (2)	0.3015 (2)	0.0626 (8)	
H14	0.3949	0.5633	0.3344	0.075*	
C15	0.4719 (4)	0.4971 (2)	0.1964 (2)	0.0560 (7)	
H15	0.4741	0.5639	0.1595	0.067*	
C16	0.5139 (3)	0.3989 (2)	0.14617 (19)	0.0429 (6)	
H16	0.5428	0.3998	0.0749	0.051*	
C17	0.5139 (2)	0.29761 (18)	0.20047 (17)	0.0330 (5)	
C18	0.6650 (3)	0.2313 (2)	−0.25469 (19)	0.0427 (6)	
C19	0.7963 (3)	0.26720 (19)	−0.14760 (19)	0.0400 (5)	
C20	0.9572 (3)	0.3118 (2)	−0.1525 (2)	0.0571 (7)	
H20	0.9828	0.3190	−0.2241	0.069*	
C21	1.0794 (3)	0.3457 (3)	−0.0539 (3)	0.0704 (9)	
H21	1.1863	0.3754	−0.0590	0.084*	
C22	1.0418 (3)	0.3351 (3)	0.0525 (2)	0.0638 (8)	
H22	1.1237	0.3575	0.1195	0.077*	
C23	0.8834 (3)	0.2913 (2)	0.0595 (2)	0.0488 (6)	
H23	0.8592	0.2850	0.1317	0.059*	
C24	0.7592 (3)	0.25648 (18)	−0.03888 (18)	0.0369 (5)	
N1	0.0201 (2)	0.19659 (14)	0.49270 (14)	0.0338 (4)	
N2	−0.0105 (2)	−0.01530 (14)	0.29787 (14)	0.0309 (4)	
O1	0.26802 (17)	0.02247 (12)	0.53842 (12)	0.0354 (3)	
H11	0.3286	0.0809	0.5256	0.042*	
H12	0.3335	−0.0201	0.5745	0.042*	
O2	0.4542 (2)	0.20761 (14)	0.47789 (13)	0.0493 (4)	
O3	0.5396 (2)	0.11191 (14)	0.33731 (13)	0.0466 (4)	
O4	0.5199 (2)	0.20487 (17)	−0.25451 (14)	0.0602 (5)	
O5	0.7208 (2)	0.23484 (17)	−0.35055 (14)	0.0606 (5)	
H51	0.6376	0.2168	−0.4033	0.073*	
S1	0.55261 (7)	0.19603 (5)	−0.03290 (5)	0.04397 (16)	
S2	0.56728 (7)	0.16800 (5)	0.13681 (5)	0.03939 (15)	

### Atomic displacement parameters (Å^2^)

**Table:**

	*U*^11^	*U*^22^	*U*^33^	*U*^12^	*U*^13^	*U*^23^
Mn1	0.0335 (2)	0.0252 (2)	0.0210 (2)	0.00591 (17)	0.00676 (18)	0.00459 (17)
C1	0.0358 (12)	0.0617 (15)	0.0222 (11)	−0.0030 (11)	0.0030 (9)	0.0019 (10)
C2	0.0351 (12)	0.0648 (16)	0.0268 (11)	−0.0044 (11)	0.0104 (9)	0.0038 (11)
C3	0.0363 (11)	0.0293 (11)	0.0213 (10)	0.0047 (9)	0.0078 (8)	0.0014 (8)
C4	0.0318 (11)	0.0534 (14)	0.0227 (11)	0.0015 (10)	0.0037 (9)	0.0031 (9)
C5	0.0346 (12)	0.0489 (13)	0.0267 (11)	0.0024 (10)	0.0097 (9)	0.0042 (9)
C6	0.0566 (15)	0.0302 (12)	0.0442 (14)	0.0031 (11)	−0.0087 (11)	0.0018 (10)
C7	0.0539 (15)	0.0344 (13)	0.0501 (15)	0.0061 (11)	−0.0135 (12)	0.0054 (11)
C8	0.0329 (11)	0.0299 (11)	0.0357 (11)	0.0025 (9)	0.0080 (9)	0.0038 (9)
C9	0.0481 (14)	0.0352 (12)	0.0362 (12)	0.0066 (10)	−0.0035 (10)	−0.0016 (10)
C10	0.0500 (14)	0.0375 (13)	0.0361 (12)	0.0132 (10)	0.0007 (11)	0.0069 (10)
C11	0.0350 (12)	0.0436 (13)	0.0301 (12)	0.0033 (10)	0.0040 (9)	0.0091 (10)
C12	0.0338 (11)	0.0401 (12)	0.0293 (11)	0.0053 (9)	0.0055 (9)	0.0079 (9)
C13	0.0643 (16)	0.0508 (15)	0.0367 (13)	0.0151 (12)	0.0183 (12)	0.0072 (11)
C14	0.095 (2)	0.0449 (15)	0.0562 (17)	0.0262 (14)	0.0249 (16)	0.0083 (13)
C15	0.0811 (19)	0.0419 (14)	0.0492 (16)	0.0164 (13)	0.0145 (14)	0.0188 (12)
C16	0.0522 (14)	0.0476 (14)	0.0323 (12)	0.0096 (11)	0.0125 (11)	0.0129 (10)
C17	0.0298 (11)	0.0399 (12)	0.0282 (11)	0.0061 (9)	0.0019 (9)	0.0069 (9)
C18	0.0582 (16)	0.0414 (13)	0.0326 (12)	0.0110 (11)	0.0155 (11)	0.0073 (10)
C19	0.0455 (13)	0.0426 (13)	0.0353 (12)	0.0120 (10)	0.0124 (10)	0.0068 (10)
C20	0.0574 (17)	0.0724 (19)	0.0480 (15)	0.0075 (14)	0.0245 (13)	0.0110 (13)
C21	0.0445 (16)	0.098 (2)	0.068 (2)	−0.0070 (15)	0.0194 (15)	0.0086 (17)
C22	0.0462 (16)	0.086 (2)	0.0505 (16)	−0.0066 (14)	0.0019 (13)	0.0022 (15)
C23	0.0469 (14)	0.0637 (16)	0.0328 (12)	0.0006 (12)	0.0069 (11)	0.0014 (11)
C24	0.0404 (12)	0.0382 (12)	0.0326 (12)	0.0045 (10)	0.0099 (10)	0.0015 (9)
N1	0.0400 (10)	0.0308 (9)	0.0303 (9)	0.0072 (8)	0.0059 (8)	0.0032 (7)
N2	0.0366 (10)	0.0330 (9)	0.0242 (9)	0.0039 (7)	0.0091 (8)	0.0032 (7)
O1	0.0333 (8)	0.0389 (8)	0.0353 (8)	0.0072 (6)	0.0059 (6)	0.0141 (6)
O2	0.0654 (11)	0.0569 (11)	0.0292 (8)	0.0068 (8)	0.0160 (8)	0.0132 (7)
O3	0.0575 (10)	0.0477 (10)	0.0425 (9)	0.0208 (8)	0.0162 (8)	0.0204 (8)
O4	0.0569 (12)	0.0838 (14)	0.0348 (9)	−0.0073 (10)	0.0084 (8)	0.0034 (9)
O5	0.0658 (12)	0.0897 (14)	0.0305 (9)	0.0164 (10)	0.0168 (8)	0.0068 (9)
S1	0.0428 (3)	0.0576 (4)	0.0289 (3)	−0.0034 (3)	0.0082 (2)	0.0014 (3)
S2	0.0464 (3)	0.0420 (3)	0.0326 (3)	0.0074 (3)	0.0131 (2)	0.0070 (2)

### Geometric parameters (Å, °)

**Table:**

Mn1—O1	2.1453 (18)	C11—C12	1.505 (3)
Mn1—O1^i^	2.1453 (18)	C12—C13	1.387 (3)
Mn1—N1^i^	2.312 (2)	C12—C17	1.407 (3)
Mn1—N1	2.312 (2)	C13—C14	1.376 (3)
Mn1—N2	2.384 (2)	C13—H13	0.9300
Mn1—N2^i^	2.384 (2)	C14—C15	1.381 (4)
C1—N2	1.341 (3)	C14—H14	0.9300
C1—C2	1.378 (3)	C15—C16	1.374 (3)
C1—H1	0.9300	C15—H15	0.9300
C2—C3	1.388 (3)	C16—C17	1.399 (3)
C2—H2	0.9300	C16—H16	0.9300
C3—C4	1.391 (3)	C17—S2	1.792 (2)
C3—C3^ii^	1.495 (4)	C18—O4	1.206 (3)
C4—C5	1.384 (3)	C18—O5	1.322 (3)
C4—H4	0.9300	C18—C19	1.487 (3)
C5—N2	1.338 (3)	C19—C20	1.390 (3)
C5—H5	0.9300	C19—C24	1.405 (3)
C6—N1	1.338 (3)	C20—C21	1.376 (4)
C6—C7	1.376 (3)	C20—H20	0.9300
C6—H6	0.9300	C21—C22	1.380 (4)
C7—C8	1.393 (3)	C21—H21	0.9300
C7—H7	0.9300	C22—C23	1.376 (4)
C8—C9	1.393 (3)	C22—H22	0.9300
C8—C8^iii^	1.495 (4)	C23—C24	1.385 (3)
C9—C10	1.378 (3)	C23—H23	0.9300
C9—H9	0.9300	C24—S1	1.794 (2)
C10—N1	1.347 (3)	O1—H11	0.8488
C10—H10	0.9300	O1—H12	0.8488
C11—O3	1.249 (3)	O5—H51	0.8220
C11—O2	1.267 (3)	S1—S2	2.0539 (14)
			
O1—Mn1—O1^i^	180.0	C17—C12—C11	121.76 (19)
O1—Mn1—N1^i^	93.78 (6)	C14—C13—C12	121.6 (2)
O1^i^—Mn1—N1^i^	86.22 (6)	C14—C13—H13	119.2
O1—Mn1—N1	86.22 (6)	C12—C13—H13	119.2
O1^i^—Mn1—N1	93.78 (6)	C13—C14—C15	119.2 (2)
N1^i^—Mn1—N1	180.0	C13—C14—H14	120.4
O1—Mn1—N2	91.06 (5)	C15—C14—H14	120.4
O1^i^—Mn1—N2	88.94 (5)	C16—C15—C14	120.5 (2)
N1^i^—Mn1—N2	93.20 (6)	C16—C15—H15	119.8
N1—Mn1—N2	86.80 (6)	C14—C15—H15	119.8
O1—Mn1—N2^i^	88.94 (5)	C15—C16—C17	121.0 (2)
O1^i^—Mn1—N2^i^	91.06 (5)	C15—C16—H16	119.5
N1^i^—Mn1—N2^i^	86.80 (6)	C17—C16—H16	119.5
N1—Mn1—N2^i^	93.20 (6)	C16—C17—C12	118.4 (2)
N2—Mn1—N2^i^	180.0	C16—C17—S2	122.06 (17)
N2—C1—C2	124.3 (2)	C12—C17—S2	119.51 (16)
N2—C1—H1	117.9	O4—C18—O5	122.9 (2)
C2—C1—H1	117.9	O4—C18—C19	123.2 (2)
C1—C2—C3	120.5 (2)	O5—C18—C19	113.9 (2)
C1—C2—H2	119.7	C20—C19—C24	118.7 (2)
C3—C2—H2	119.7	C20—C19—C18	121.1 (2)
C2—C3—C4	115.27 (18)	C24—C19—C18	120.2 (2)
C2—C3—C3^ii^	122.7 (2)	C21—C20—C19	121.6 (2)
C4—C3—C3^ii^	122.0 (2)	C21—C20—H20	119.2
C5—C4—C3	120.80 (19)	C19—C20—H20	119.2
C5—C4—H4	119.6	C20—C21—C22	119.3 (3)
C3—C4—H4	119.6	C20—C21—H21	120.3
N2—C5—C4	123.71 (19)	C22—C21—H21	120.3
N2—C5—H5	118.1	C23—C22—C21	120.1 (3)
C4—C5—H5	118.1	C23—C22—H22	119.9
N1—C6—C7	124.6 (2)	C21—C22—H22	119.9
N1—C6—H6	117.7	C22—C23—C24	121.2 (2)
C7—C6—H6	117.7	C22—C23—H23	119.4
C6—C7—C8	120.3 (2)	C24—C23—H23	119.4
C6—C7—H7	119.8	C23—C24—C19	119.0 (2)
C8—C7—H7	119.8	C23—C24—S1	122.39 (17)
C7—C8—C9	115.4 (2)	C19—C24—S1	118.59 (17)
C7—C8—C8^iii^	121.8 (2)	C6—N1—C10	114.97 (18)
C9—C8—C8^iii^	122.8 (2)	C6—N1—Mn1	121.51 (14)
C10—C9—C8	120.5 (2)	C10—N1—Mn1	123.27 (14)
C10—C9—H9	119.8	C5—N2—C1	115.39 (17)
C8—C9—H9	119.8	C5—N2—Mn1	126.19 (13)
N1—C10—C9	124.1 (2)	C1—N2—Mn1	118.17 (13)
N1—C10—H10	118.0	Mn1—O1—H11	124.9
C9—C10—H10	118.0	Mn1—O1—H12	127.9
O3—C11—O2	124.6 (2)	H11—O1—H12	106.9
O3—C11—C12	117.53 (19)	C18—O5—H51	105.1
O2—C11—C12	117.8 (2)	C24—S1—S2	105.21 (8)
C13—C12—C17	119.2 (2)	C17—S2—S1	104.13 (8)
C13—C12—C11	118.9 (2)		
			
N2—C1—C2—C3	−0.7 (4)	C20—C19—C24—C23	0.4 (3)
C1—C2—C3—C4	−1.1 (3)	C18—C19—C24—C23	−179.5 (2)
C1—C2—C3—C3^ii^	179.9 (2)	C20—C19—C24—S1	−178.70 (18)
C2—C3—C4—C5	1.3 (3)	C18—C19—C24—S1	1.4 (3)
C3^ii^—C3—C4—C5	−179.7 (2)	C7—C6—N1—C10	−4.3 (4)
C3—C4—C5—N2	0.3 (3)	C7—C6—N1—Mn1	170.2 (2)
N1—C6—C7—C8	1.4 (4)	C9—C10—N1—C6	4.3 (3)
C6—C7—C8—C9	1.6 (4)	C9—C10—N1—Mn1	−170.04 (18)
C6—C7—C8—C8^iii^	−178.1 (3)	O1—Mn1—N1—C6	137.19 (18)
C7—C8—C9—C10	−1.6 (3)	O1^i^—Mn1—N1—C6	−42.81 (18)
C8^iii^—C8—C9—C10	178.1 (2)	N1^i^—Mn1—N1—C6	−123 (100)
C8—C9—C10—N1	−1.5 (4)	N2—Mn1—N1—C6	45.92 (18)
O3—C11—C12—C13	−176.1 (2)	N2^i^—Mn1—N1—C6	−134.08 (18)
O2—C11—C12—C13	3.0 (3)	O1—Mn1—N1—C10	−48.84 (17)
O3—C11—C12—C17	−0.6 (3)	O1^i^—Mn1—N1—C10	131.16 (17)
O2—C11—C12—C17	178.5 (2)	N1^i^—Mn1—N1—C10	51 (100)
C17—C12—C13—C14	−1.8 (4)	N2—Mn1—N1—C10	−140.12 (18)
C11—C12—C13—C14	173.8 (2)	N2^i^—Mn1—N1—C10	39.88 (18)
C12—C13—C14—C15	−0.5 (4)	C4—C5—N2—C1	−2.0 (3)
C13—C14—C15—C16	1.8 (4)	C4—C5—N2—Mn1	172.24 (16)
C14—C15—C16—C17	−0.9 (4)	C2—C1—N2—C5	2.2 (3)
C15—C16—C17—C12	−1.4 (3)	C2—C1—N2—Mn1	−172.51 (19)
C15—C16—C17—S2	179.74 (19)	O1—Mn1—N2—C5	176.09 (17)
C13—C12—C17—C16	2.7 (3)	O1^i^—Mn1—N2—C5	−3.91 (17)
C11—C12—C17—C16	−172.80 (19)	N1^i^—Mn1—N2—C5	82.25 (17)
C13—C12—C17—S2	−178.41 (17)	N1—Mn1—N2—C5	−97.75 (17)
C11—C12—C17—S2	6.1 (3)	N2^i^—Mn1—N2—C5	−142 (100)
O4—C18—C19—C20	−171.1 (2)	O1—Mn1—N2—C1	−9.86 (16)
O5—C18—C19—C20	6.7 (3)	O1^i^—Mn1—N2—C1	170.14 (16)
O4—C18—C19—C24	8.8 (4)	N1^i^—Mn1—N2—C1	−103.70 (16)
O5—C18—C19—C24	−173.4 (2)	N1—Mn1—N2—C1	76.30 (16)
C24—C19—C20—C21	−0.2 (4)	N2^i^—Mn1—N2—C1	33 (100)
C18—C19—C20—C21	179.8 (3)	C23—C24—S1—S2	−9.7 (2)
C19—C20—C21—C22	0.1 (5)	C19—C24—S1—S2	169.35 (16)
C20—C21—C22—C23	−0.2 (5)	C16—C17—S2—S1	−15.88 (19)
C21—C22—C23—C24	0.4 (4)	C12—C17—S2—S1	165.29 (15)
C22—C23—C24—C19	−0.5 (4)	C24—S1—S2—C17	91.50 (11)
C22—C23—C24—S1	178.6 (2)		

Symmetry codes: (i) −*x*, −*y*, −*z*+1; (ii) −*x*, −*y*, −*z*; (iii) −*x*, −*y*+1, −*z*+1.

### Hydrogen-bond geometry (Å, °)

**Table:**

*D*—H···*A*	*D*—H	H···*A*	*D*···*A*	*D*—H···*A*
O1—H11···O2	0.85	1.92	2.761 (3)	173
O1—H12···O3^iv^	0.85	1.82	2.667 (3)	174
O5—H51···O2^v^	0.82	1.83	2.637 (3)	169
C4—H4···S1^vi^	0.93	2.86	3.562 (3)	133
C23—H23···S2	0.93	2.66	3.191 (3)	117
C22—H22···Cg1^vii^	0.93	2.94	3.79 (2)	153

Symmetry codes: (iv) −*x*+1, −*y*, −*z*+1; (v) *x*, *y*, *z*−1; (vi) −*x*, −*y*, −*z*; (vii) *x*+1, *y*, *z*.
